# Layer-by-Layer Repair of Small-Scale Damage of Fused Silica Based on the Magnetorheological Method

**DOI:** 10.3390/mi12101233

**Published:** 2021-10-10

**Authors:** Mingjie Deng, Ci Song, Feng Shi, Wanli Zhang, Ye Tian, Guipeng Tie

**Affiliations:** 1 College of Intelligence Science and Technology, National University of Defense Technology, Changsha 410073, China; dengmingjie19@163.com (M.D.); sf.wind@yahoo.com (F.S.); zhangwanli17@nudt.edu.cn (W.Z.); tianyecomeon@sina.cn (Y.T.); tieguipeng@163.com (G.T.); 2 Hunan Key Laboratory of Ultra-Precision Machining Technology, National University of Defense Technology, Changsha 410073, China; 3 Laboratory of Science and Technology on Integrated Logistics Support, National University of Defense Technology, Changsha 410073, China

**Keywords:** fused silica, small-scale damage, magnetorheological repair, morphology, fluorescence, photothermal absorption

## Abstract

The magnetorheological (MR) repair method can effectively repair the small-scale damage of fused silica optics and further improve the laser-induced damage threshold of fused silica optics. However, at present, the rules of MR repair of small-scale damage of fused silica are not clear and cannot provide further guidance for the repair process. In this paper, the fused silica damage samples were repaired layer by layer by the MR method. The number and size changes of all the surface damage, the morphology, the fluorescence area distribution, and photothermal-absorption value of a single typical small-scale damage were measured. Through dark field scattering imaging, it is found that when the repair depth is 5 μm, the repair completion rate of damage with a transverse size less than 50 μm can reach 44%, and the repair efficiency decreases gradually with the repair process. Focusing on the whole repair process of a single typical, small-scale damage—due to the flexible shear removal mechanism of the MR method—the repair process of damage can be divided into three stages, which as a whole is a top-down, from outside to inside process. The first stage is the process of removing the surface of the damage layer by layer. In this process, MR fluid will introduce pollution to the inside of the damage. In the second stage, MR fluid begins to repair the inside of the damage. In the third stage, the MR ribbon completely covers the inside of the damage, and the repair effect is the most obvious. The measurement results of photothermal absorption and fluorescence area distribution of damage confirm this process. The photothermal absorption value and fluorescence area distribution of damage do not simply decrease with the repair process. On the contrary, they gradually increase first, and then decrease significantly when the damage depth reaches less than 1 μm. As the thickness of the MR ribbon is 1 μm, the reduction in the photothermal absorption value and fluorescence area of the damage is due to the process of repairing the inside of the damage. The results show that the absorbent impurities inside the small-scale damage of fused silica are the main factor affecting the performance. The key to repairing the small-scale damage of fused silica by the MR method is that the damaged interior must be repaired effectively. This paper outlines the MR repair method of small-scale damage of fused silica, which is of great significance to optimize the MR repair process.

## 1. Introduction

The elements of fused silica in high-power laser systems are required to be precise, which not only requires excellent optical indexes such as high surface accuracy and low surface roughness, but also puts forward strict requirements for the performance of laser energy loaded on the elements [1]. The research shows that the laser energy gathered by the surface and sub surface defects of fused silica is the main reason for the reduction of laser damage resistance of the fused silica. [2] These small defects grow destructively and dramatically under the high-power laser, resulting in permanent failure of optical elements. According to the standard of the National Ignition Facility (NIF), if the damage area on the optical element accounts for 3% of the total area, it is deemed that the element has reached its service life and must be replaced. [3]

In order to improve the utilization rate of fused silica components and overcome the problems of high demand, a difficult manufacturing process, and the easily damaged surface of fused silica, the method of reusing damaged components after repair is proposed. [4] At present, the repair methods for the damage of fused silica components mainly include CO_2_ laser repair [5], hydrofluoric acid etching [6], plasma etching, and micro flame processing. Among them, CO_2_ laser repair has achieved a good repair effect and has become the main means of repairing optical elements; [7,8] however, the current repair technology has some limitations. The main repair object is large-scale individual damage, ignoring the frequent small-scale cluster damage. [9] A previous study shows that the probability of small-scale damage of clusters with transverse size less than 50 μm is higher than 95%, and the probability of increased damage in the subsequent process is great. [10] At present, there are few studies on the repair of small-scale cluster damage.

The National University of Defense Technology [11,12] has proposed a method to repair the damage of fused silica by the MR method, which can effectively repair the small-scale cluster damage of fused silica elements in high-power laser systems, and the repair success rate can reach more than 90%. After a series of post-treatment processes, the elements can be basically restored to the level before damage. However, the repair process in this study is cumbersome and time-consuming. It does not fully and deeply reveal the process of MR repair, and there is a lack of research on the guidelines of repairing a single small-scale damage. There are still many unknown factors in the process of MR repair and further optimization is needed.

In this paper, the fused silica optic was repaired by the MR method. The number and size of surface damage area, the morphology, the fluorescence area distribution, and the photothermal absorption value of typical small-scale damage were systematically measured and analyzed, and the evolution guidelines of typical damage repaired by MR were studied. It was found that the absorbent impurities inside the small-scale damage of fused silica is the main factor affecting the performance. The key to repairing the small-scale damage of fused silica by the MR method is that MR fluid must effectively repair the damaged interior. This paper reveals the mechanism of MR repair of fused silica damage, which is of great significance to optimize the MR repair process.

## 2. Experiment

A fused silica sample with a size of 50 mm × 50 mm × 10 mm and made of Heraeus 312 was used. The surface roughness of the element was 0.975 nm, which meets the requirements of the actual system, and the surface roughness was less than 1nm. The fused silica element was irradiated by a pulsed laser with a wavelength of 355 nm and a pulse width of 7 ns through an S-shaped scanning path to prepare the damaged sample. The average laser output energy was 65 mJ, the 1/e^2^ spot radius was 0.5 cm, and the calculated energy density was about 8.28 J/cm^2^.

The KDUPF-700 MRF, which is made by National University of Defense Technology, Changsha, China, was used to repair the optic layer by layer. The repair depth was 1 μm each time and the process parameters are shown in Table 1. In the experiment, MR repairing the surface damage produced obvious tails and destroyed the surface quality. Therefore, the machining direction was kept unchanged in the actual process to minimize the damage diffusion.

Optics were measured after each repair. We used the laser scattering defect detector of super-smooth surfaces—which is made by ZC Optoelectronic Technologies, Ltd., Hefei, China—to detect the surface damage of the sample, so as to track the evolution of the number and size of the damage in the repair process. The morphology and contour of the damage were observed by an icon atomic force microscope produced by Bruker Corporation, Billerica, MA, USA. The photothermal absorption value and fluorescence area of fused silica surface damage were measured in situ by using the nano damage precursor multi-modal detector produced by ZC Optoelectronic Technologies, Ltd.

## 3. Result and Discussion

### 3.1. Evolution of the Number and Size of Damage

**Figure 1:**
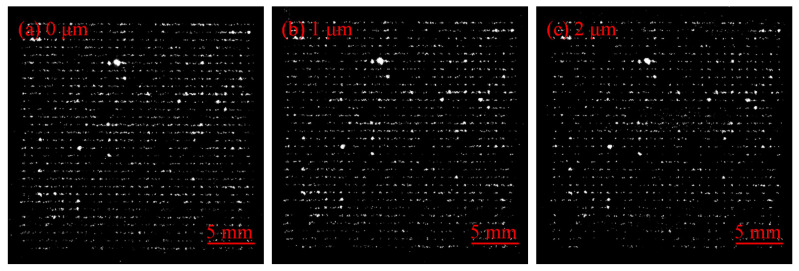

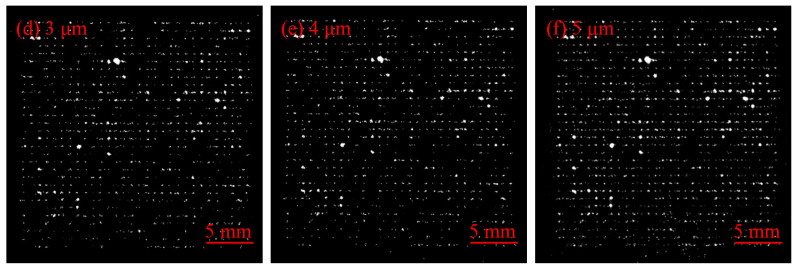
Dark field images of damage at different repair depths: (**a**) initial; (**b**) repair depth is 1 μm; (**c**) repair depth is 2 μm; (**d**) repair depth is 3 μm; (**e**) repair depth is 4 μm; (**f**) repair depth is 5 μm.

**Figure 2:**
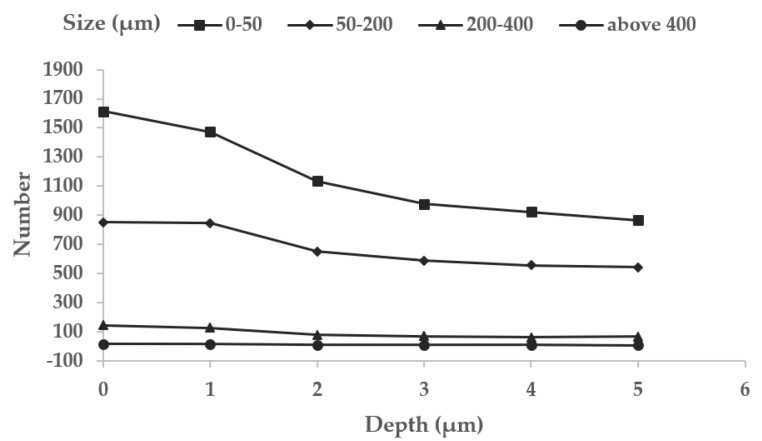
Evolution of the number and size of damages with repair depth.

In order to study the evolution of the number and size of damaged areas in the repair process of fused silica, the layer-by-layer MR repair method is adopted, and the surface damage of the sample is measured by the laser scattering defect detector of super-smooth surfaces. The dark field micro scattering images of fused silica surface damage at different repair depths are shown in Figure 1. Based on the scattering image, the number and size of fused silica surface damage at different repair depths are statistically analyzed by the image processing method. The results are shown in Figure 2. The results reflect the overall characteristics of the MR repair of the fused silica damage. Before the MR repair, the number of damages below 50 μm accounted for 61.5% of the total damage. The number of damages decreased with the increase of repair depth. After the repair depth reached 5 μm, the damage below 50 μm accounted for 58.5% of the total damage, and the total number of damages decreased from 2628 to 1472, a decrease of 44%. The results show that the damaged areas smaller than 50 μm can be effectively repaired by the MR method.

### 3.2. Evolution of the Damage Morphology

**Figure 3:**
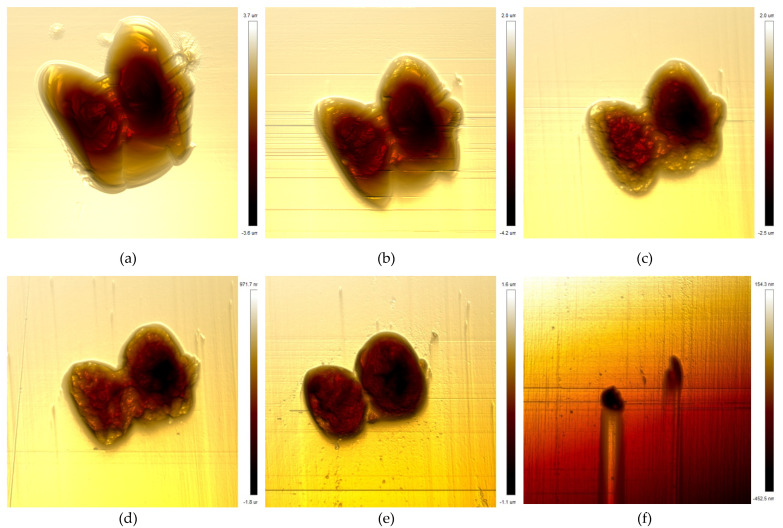
The images of damage morphology: (**a**) initial, 1272 nm RMS; (**b**) repair depth is 1 μm, 971 nm RMS; (**c**) repair depth is 2 μm, 610 nm RMS; (**d**) repair depth is 3 μm, 373 nm RMS; (**e**) repair depth is 4 μm, 374 nm RMS; (**f**) repair depth is 5 μm, 20.9 nm RMS.

**Figure 4:**
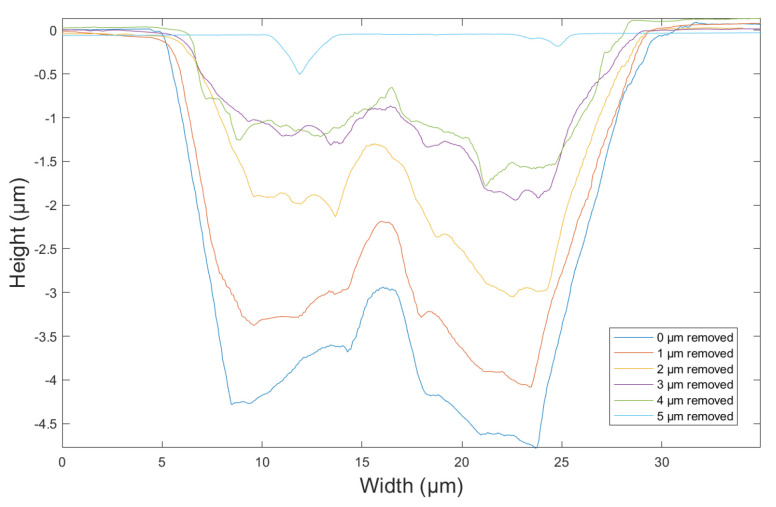
The graphs of damage profile structure.

The study shows that the damage points with a transverse size greater than 20 μm still have a probability of more than 60% to continue to grow. [10] When fused silica is irradiated by a fundamental frequency laser, due to light field modulation and other reasons, the plasma temperature inside the damage rises sharply, and the transverse size of the damage point increases exponentially with the irradiation pulse. [13] The specific equation is:

D = D_0_ ∗ exp (λ ∗ N) (1)

where D is the transverse size of the damage point, D_0_ is the initial size, N is the number of irradiation pulses, and λ is the growth coefficient. Reducing the transverse size and depth of damage points is of great significance to improve the damage threshold. As the evolution trend of small-scale damage is similar, in order to study the morphology evolution of small-scale damage in the repair process, a typical damage with a transverse size of less than 50 μm was selected as the research object. In order to quantitatively analyze the evolution of morphology, the damage was measured in situ many times by an atomic force microscope. The measurement results are shown in Figure 3, and the section depth of damage is shown in Figure 4. The AFM image is 40μm × 40μm.

The results show that the initial transverse size of the damage was 35 μm and the depth was 4.8 μm, which belongs to a typical small-scale damage smaller than 50 μm. According to the changes of damage morphology and profile, the repair process is roughly divided into three stages. The first stage is for the repair depth of 0–2 μm. It can be seen that the transverse size of the damage decreases gradually, and the interior of the damage basically does not change. This stage is mainly the process of removing the damaged surface layer by layer, and the interior of the damage is basically not affected, mainly because the MR ribbon is not thick enough to contact the interior of the damage. The second stage is for the repair depth of 2–4 μm. In this stage, the transverse size decreased and the profile began to change, which indicates that the MR method not only removes the damaged surface, but also begins contact with the interior. However, due to different pressure depth, the surface removal rate was higher than the internal removal rate, and the repair process was still continuing. The repair depth of 4–5 μm is the third stage. In this stage, the repair depth has exceeded the damage depth, and the whole repair process of small-scale damage has been basically completed. Throughout the whole repair process, due to the flexible shear removal mechanism of the MR method, the repair of damage by MR is actually a top-down, from the outside to the inside process, and only when the MR ribbon contacts the inside of the damage will MR have a repair effect on the inside of the damage.

### 3.3. Evolution of Fluorescence Area

Laser induced fluorescence imaging is a new nondestructive testing method based on the principle of “absorption transition emission” to obtain the spatial distribution information of optical characteristic substances. [14] The results show that under the excitation of specific wavelength laser, the material information with absorptivity and emissivity such as polluting defects produced by polishing and plasma formed in the process of damage can be effectively characterized.

**Figure 5:**
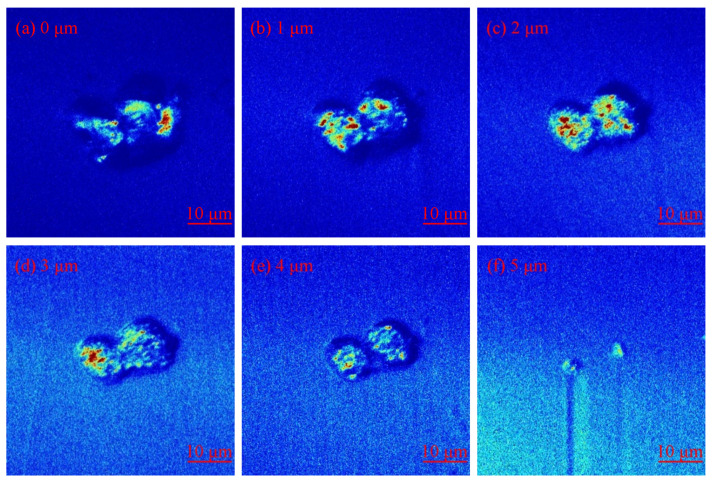
The images of damage fluorescence distribution: (**a**) initial; (**b**) repair depth is 1μm; (**c**) repair depth is 2 μm; (**d**) repair depth is 3 μm; (**e**) repair depth is 4 μm; (**f**) repair depth is 5 μm.

**Figure 6:**
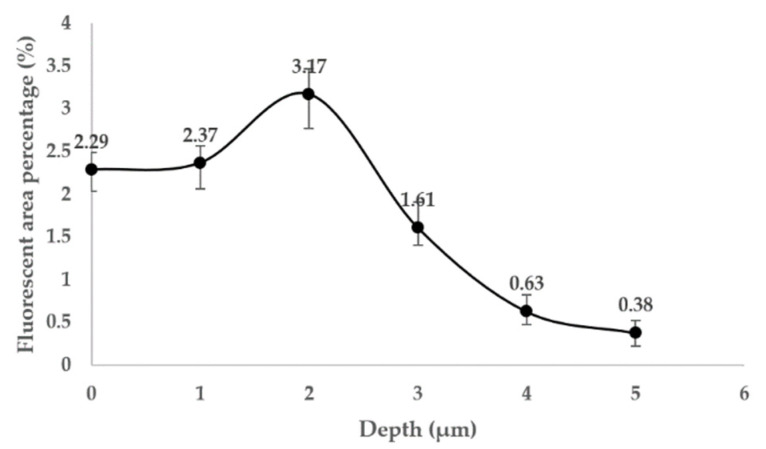
The percentage of damage fluorescent area.

In order to study the evolution of fluorescence information inside the damage during MR repair, the fluorescence distribution inside the damage of fused silica was measured in situ by the nano damage precursor multi-modal detector. The results are shown in Figure 5. In order to quantify the fluorescence detection results, image preprocessing is performed on the detection results, and the fluorescence area percentage of damage is calculated. Figure 6 shows the quantitative statistical results of fluorescence area of damage.

The results show that there are absorbent fluorescent substances in the damage before the repair. When the repair depth is 0–2 μm, the fluorescence area inside the damage increases gradually until it is the strongest. According to the analysis in the previous section, at this time, the main function of MR is to remove the damaged surface of fused silica, and the interior of the damage is basically not affected. On the contrary, the fluorescent area increases due to the fluorescent impurities introduced by the MR fluid. After the repair depth reached 2 μm, the fluorescence area decreased rapidly to a very low level. At this time, it was in the second and third stage of MR repair. Compared with the introduction of fluorescent impurities, MR has a more obvious effect on the internal repair of damage; thus, the fluorescent area is greatly reduced.

### 3.4. Evolution of Photothermal Absorption

The temperature rise caused by the heat absorption of pollutants on the surface of fused silica and the local modulation of incident light field are the main inducements for damage growth. To some extent, the behavior mechanism of laser-induced surface damage growth of fused silica can be attributed to the influence of surface absorption properties. In order to study the evolution of typical damage absorption properties of fused silica in the process of MR repair, the damage was detected in situ by the nano damage precursor multi-modal detector. The test area was 80 μm × 80 μm. The measured average absorption value in this area represents the average absorption level of damage, and the maximum absorption value in this area represents the highest absorption level of damage, so as to quantitatively reflect the evolution of photothermal absorption of damage in the process of MR repair.

**Figure 7:**
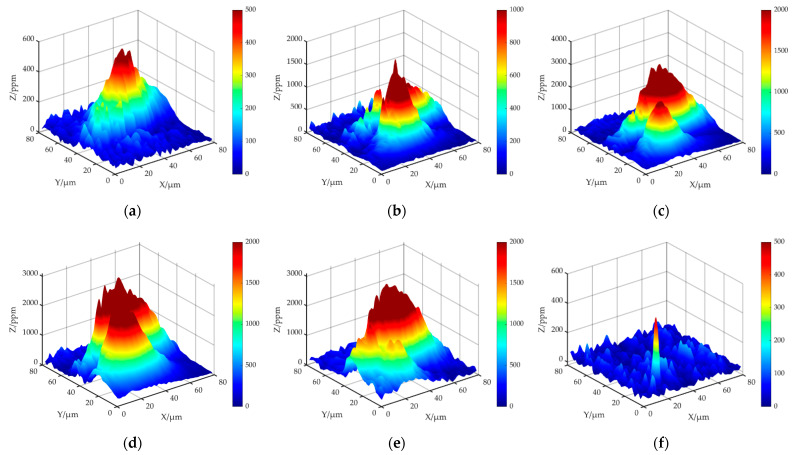
The images of damage photothermal absorption: (**a**) initial; (**b**) repair depth is 1 μm; (**c**) repair depth is 2 μm; (**d**) repair depth is 3 μm; (**e**) repair depth is 4 μm; (**f**) repair depth is 5 μm.

**Figure 8:**
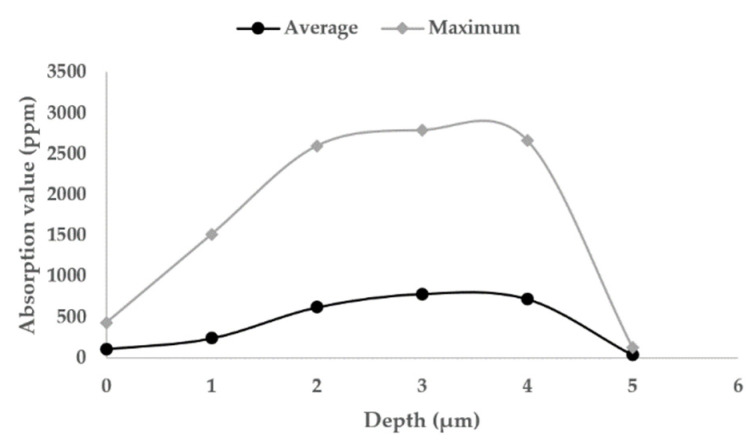
The peak and average absorption of damage.

Figure 7 shows the two-dimensional results of photothermal absorption of damage at different repair depths. Figure 8 shows the quantitative statistical results of the corresponding average and maximum photothermal absorption. Analyzing the absorption curve, it was found that the photothermal absorption value of damage does not simply decrease with the repair process, but increases first and then decreases. When the repair depth was 0–2 μm, the photothermal absorption value of the damage increased rapidly. At this time, it is the first stage of MR repair of the damage and MR introduces a lot of pollution into the damage. When the repair depth was 2–4 μm, it was in the second stage of MR repair of damage. At this time, there is not only the repair of internal damage, but also the process of introducing pollutants by the MR fluid is occurring, and the absorption was basically maintained at a certain level. When the repair depth reaches the last 1 μm, the MR ribbon can repair the entire damaged interior, and the absorption value decreases in a cliff like manner. With the complete repair of the damage, the absorption value was very close to the expected goal.

## 4. Conclusions

In this paper, the fused silica damaged optics were repaired layer by layer by the MR process. The overall number and size changes of the surface damage and the evolution laws of morphology, the photothermal absorption value, and the fluorescence distribution of typical small-scale damage were systematically measured. The mechanism of repairing small-scale damage of fused silica by the MR method was studied in depth.

The results show that the small-scale damage of fused silica and the internal absorbent impurities are the main factors affecting the performance of the optic. Therefore, the key to repair the small-scale damage of fused silica by the MR method is that the damage must be effectively repaired by the MR method, that is, the damage depth after repair must be lower than the thickness of the MR ribbon.

## Figures and Tables

**Table 1:** Parameters of the MR process.

Item	Level
Wheel speed (r/min)	260
Flow rate (L/min)	120
Current (A)	8
Ribbon penetration depth (mm)	0.2

## Data Availability

The data presented in this study are available on request from the corresponding author. The data are not publicly available due to the data also forms part of an ongoing study.
